# Cleavable ester linked magnetic nanoparticles for labeling of solvent exposed primary amine groups of peptides/proteins

**DOI:** 10.1016/j.dib.2015.05.025

**Published:** 2015-06-18

**Authors:** Ujwal S. Patil, Laura Osorno, Angela Ellender, Casey Grimm, Matthew A Tarr

**Affiliations:** a Department of Chemistry, University of New Orleans, 2000 Lakeshore Drive, New Orleans, LA 70148, USA; b Southern Regional Research Center, 1100 Robert E. Lee Blvd., New Orleans, LA 70124, USA

## Abstract

Covalent labeling of solvent exposed amino acid residues using chemical reagents/crosslinkers followed by mass spectrometric analysis can be used to determine the solvent accessible amino acids of a protein. A variety of chemical reagents containing cleavable bonds were developed to label abundantly found lysine residues on the surface of protein. To achieve efficient separation of labeled peptides prior to mass spectrometric analysis, magnetic nanoparticles can be decorated with amino acid reactive functional groups and utilized for quick recovery of labeled peptides. [1] In this work, iron oxide magnetic nanoparticles (Fe_3_O_4_ MNPs) were synthesized by thermal decomposition method and coated with silica (SiO_2_@Fe_3_O_4_ MNPs) by reverse micro emulsion approach. The Fe_3_O_4_ MNPs and SiO_2_@Fe_3_O_4_ MNPs were characterized by TEM and XRD. The SiO_2_@Fe_3_O_4_ MNPs were further coated with amine groups and conjugated to *N*-hydroxysuccinimidyl (NHS) ester groups via a cleavable ester bond. Fluorescence based qualitative analysis of ester linked NHS ester modified SiO_2_@Fe_3_O_4_ MNPs was performed to confirm the presence of NHS ester group. The active NHS ester sites on the surface of SiO_2_@Fe_3_O_4_ MNPs were determined by depletion approach and found to be 694 active sites per 1 mg of SiO_2_@Fe_3_O_4_ MNPs. Free amine groups of a small peptide, ACTH (4–11) were labeled by ester linked, NHS ester modified SiO_2_@Fe_3_O_4_ MNPs under physiological conditions. Superparamagnetic nature of SiO_2_@Fe_3_O_4_ MNPs allowed quick and efficient magnetic separation of labeled peptides from the solution. The ester bond was further cleaved to separate labeled peptides followed by mass spectrometric analysis. The ester linked, NHS ester modified SiO_2_@Fe_3_O_4_ MNPs introduced a mass shift of 115.09 Da on amine groups of ACTH (4–11), which was confirmed by mass spectrometry.

## 1 Specifications table

**Table:** 

Subject area	Biochemistry, Materials chemistry

More specific subject area	Surface Proteomics
Type of data	Text file, figure
How data was acquired	X-ray diffraction, mass spectrometry, and fluorescence spectroscopy
Data format	Analyzed
Experimental factors	The SiO_2_@Fe_3_O_4_ MNPs were modified with NHS ester groups via a cleavable ester bond. Quantitative fluorometric characterization of ester linked NHS ester modified SiO_2_@Fe_3_O_4_ MNPs was performed to determine active NHS ester sites on the surface of SiO_2_@Fe_3_O_4_ MNPs. Solvent exposed free amine residues of peptides were labeled using cleavable ester linked NHS ester linked silica coated iron oxide magnetic nanoparticles. The labeling reaction was performed under physiological conditions to preserve the native structure of proteins. The ester bond was subsequently cleaved followed by magnetic separation of nanoparticles
Experimental features	The label generated on the solvent exposed free amine groups of peptides and proteins were identified by mass spectrometric analysis.
Data source location	New Orleans, Louisiana, USA
Data accessibility	Data is included in this article

## 2 Value of the data

• The surface exposed amine groups of peptides can be determined by labeling with ester linked NHS ester modified SiO_2_@Fe_3_O_4_ MNPs under physiological conditions.
• Cleavable ester linked NHS ester modified SiO_2_@Fe_3_O_4_ MNPs provide an effective approach to magnetically separate the labeled peptides from the solution without adding extra step of purification.
• The flurometric quantification of active NHS ester sites on the surface of SiO_2_@Fe_3_O_4_ MNPs can allow quantitative control over the labeling reaction.

## 3 Data, experimental design and methods

The data shown here is divided into four major steps: a) synthesis and characterization of SiO_2_@Fe_3_O_4_ MNPs, b) synthesis of ester linked NHS ester modified SiO_2_@Fe_3_O_4_ MNPs, c) fluorometric quantification of active NHS ester sites on the surface of SiO_2_@Fe_3_O_4_ MNPs and, d) labeling and identification of primary amine groups of ACTH (4–11) using ester linked NHS ester modified SiO_2_@Fe_3_O_4_ MNPs.

## 4 Materials and methods

Iron (III) oxyhydroxide (FeO(OH)), oleic acid, dimethyl sulfoxide (DMSO, anhydrous), (3-aminopropyl)triethoxysilane (APTES, 95%), tetraethylorthosilicate (TEOS, 99%), Igepal CO-520, cyclohexane, dansylcadaverine, ≥97%, hydroxylamine hydrochloride, bovine serum albumin (BSA) and β-lactoglobulin were purchased from Sigma-Aldrich (St. Louis, MO). 1-Octadecene was purchased from Alfa Aesar (Ward Hill, MA). Ethylene glycolbis(succinimidylsuccinate) (EGS, +99%) was purchased from ProteoChem (Loves Park, IL). Phosphate buffered saline (PBS) was purchased from Calbiochem (Billerica, MA). Dithiothreitol and iodoacetamide were purchased from Piercenet, Thermo Scientific (Rockford, IL). ACTH (4–11) was purchased from American Peptide Company (Sunnyvale,CA). Ethanol was purchased from Pharmco-AAPER (Brookefield,CT). Nanopure, deionized and distilled water (18.2 MΩ) was used for all experiments. Fluorescence measurements were performed by using Agilent Cary Eclipse fluorescence spectrophotometer (Santa Clara, CA).

## 5 Experimental design and data

### 5.1 Synthesis of iron oxide nanoparticles

**Fig. 1:**
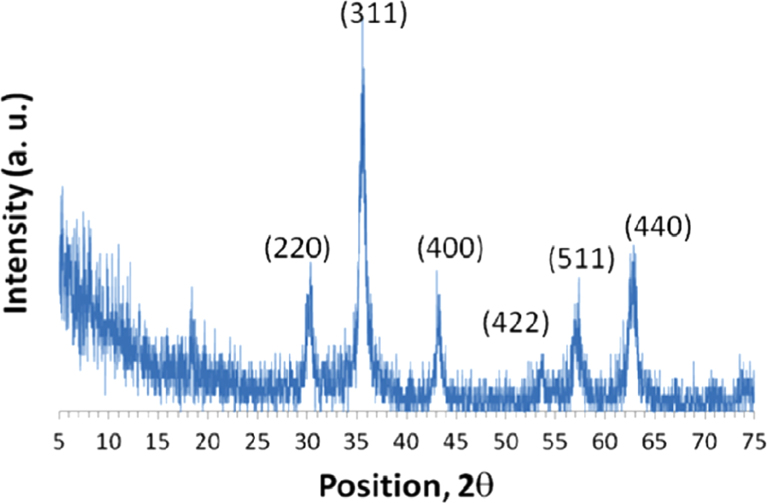
XRD data of Fe_3_O_4_ MNPs.

Iron oxide nanoparticles were synthesized as described [2] and characterized by XRD (Fig. 1).

### 5.2 Silica coating of Fe_3_O_4_ MNPs by reverse micro-emulsion approach (scheme 1)

Silica coating was performed as reported earlier. [3] The Fe_3_O_4_ MNPs (400 µL of 10 mg/mL) were dissolved in cyclohexane (4 mL) and Igepal-CO-520 (0.247 g) then sonicated for 15 min. The suspension was further mixed with tetraethyorthosilicate (25 µL) and sonicated again for 10 min. In the last step, ammonium hydroxide (50 µL) was added and sonicated for 15 min. The suspension was stirred using a magnetic stirrer at room temperature for 24 h. The SiO_2_@Fe_3_O_4_ MNPs were magnetically recovered, washed using ethanol several times, and dried at room temperature.

**Scheme 1:**
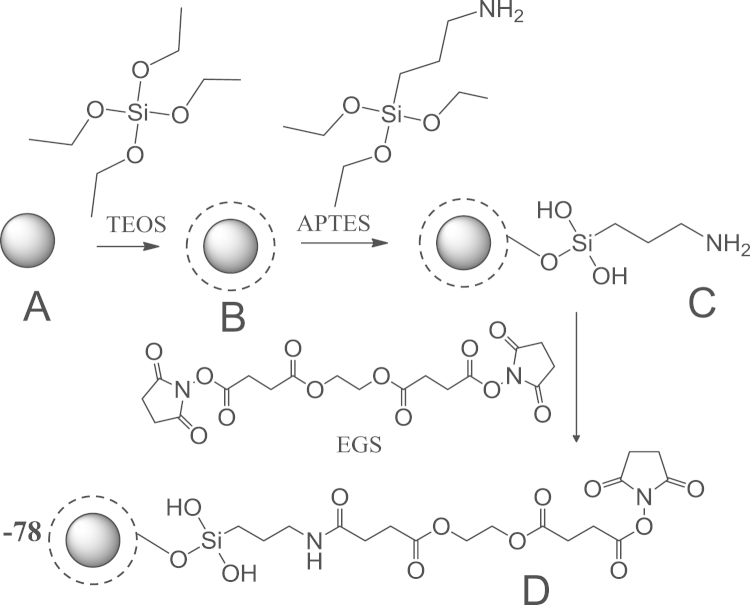
.

Scheme 1: synthesis of cleavable ester linked, NHS ester modified SiO_2_@Fe_3_O_4_ MNPs

### 5.3 Functionalization of SiO_2_@Fe_3_O_4_ MNPs with amine groups (scheme 1)

Amine groups were introduced on the surface of SiO_2_@Fe_3_O_4_ MNPs using amine containing silane coupling reagent [4]. The SiO_2_@Fe_3_O_4_ MNPs (10 mg) were resuspended in ethanol (10 mL) followed by 20 min of sonication. In the next step, APTES (95%, 100 μL) was added dropwise and the mixture was mechanically stirred at room temperature for 24 h. Amine modified Fe_3_O_4_ SiO_2_@Fe_3_O_4_ MNPs were magnetically separated, washed several times with ethanol, and air dried at room temperature.

### 5.4 Synthesis of cleavable linked, NHS ester modified SiO_2_@Fe_3_O_4_ MNPs (scheme 1)

Amine modified SiO_2_@Fe_3_O_4_ MNPs (1 mg) were mixed with ethanol (100 μL), followed by sonication for 10 min. EGS (12 mg in μL DMSO, 100 final conc. 0.13 M) was added dropwise to the solution of amine modified SiO_2_@Fe_3_O_4_ MNPs and allowed to react for 20 min at room temp. EGS modified SiO_2_@Fe_3_O_4_ MNPs were recovered by magnetic separation, washed with ethanol, and dried under vacuum.

Presence of NHS ester was determined by conjugating dansylcadaverine and mesuring the fluoscence of dansylcadaverine conjugated SiO_2_@Fe_3_O_4_ MNPs.

### 5.5 Labeling amine groups of peptides/proteins using cleavable ester linked, NHS ester modified SiO_2_@Fe_3_O_4_ MNPs

**Fig. 2:**
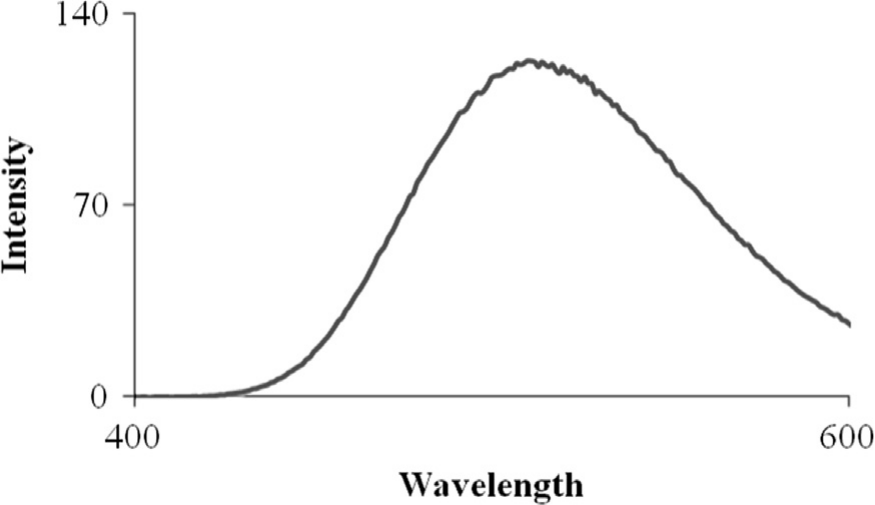
Fluorescence spectra of dansylcadaverine conjugated SiO_2_@Fe_3_O_4_ MNPs.

Labeling of ACTH (4–11), BSA and β-lactoglobulin was performed by following a protocol as reported earlier [5] with minor modifications. Protein sample (BSA or β-lactoglobulin, 10 μL, 10 mg/mL) solution was mixed with ester cleavable, NHS ester modified SiO_2_@Fe_3_O_4_ MNPs followed by addition of PBS, pH=7.4 (190 μL). The mixture was allowed to stir at room temperature for 40 min. Protein conjugated SiO_2_@Fe_3_O_4_ MNPs were magnetically separated and washed with water (6X). The unreacted NHS ester groups on the surface of SiO_2_@Fe_3_O_4_ MNPs were quenched by reacting with Tris–HCl (100 μL of 50 mM) for 15 min followed by washing with water (3X). Protein conjugated SiO_2_@Fe_3_O_4_ MNPs were incubated with urea (8 M, aq. 80 μL) and DTT (5 μL of 200 mM) at 45˚C for 1 h. Free thiol groups were alkylated with iodoacetamide (10 μL of 200 mM) for 1 h in the dark. Trypsin was added (6 μg) with ammonium bicarbonate (1 mL of 50 mM) followed by digestion for 15 h at 37 °C. The tryptic peptide conjugated SiO_2_@Fe_3_O_4_ MNPs were magnetically separated and washed with water (3X), and water:ACN (30:70, 6X). Labeled tryptic peptides were isolated from SiO_2_@Fe_3_O_4_ MNPs by cleaving the ester bond using hydroxylamine (200 μL of 2 M, pH=8.5) for 4 h at 37 °C. The SiO_2_@Fe_3_O_4_ MNPs were magnetically separated, and the supernatant was saved for further analysis Fig. 2.

**Fig. 3:**
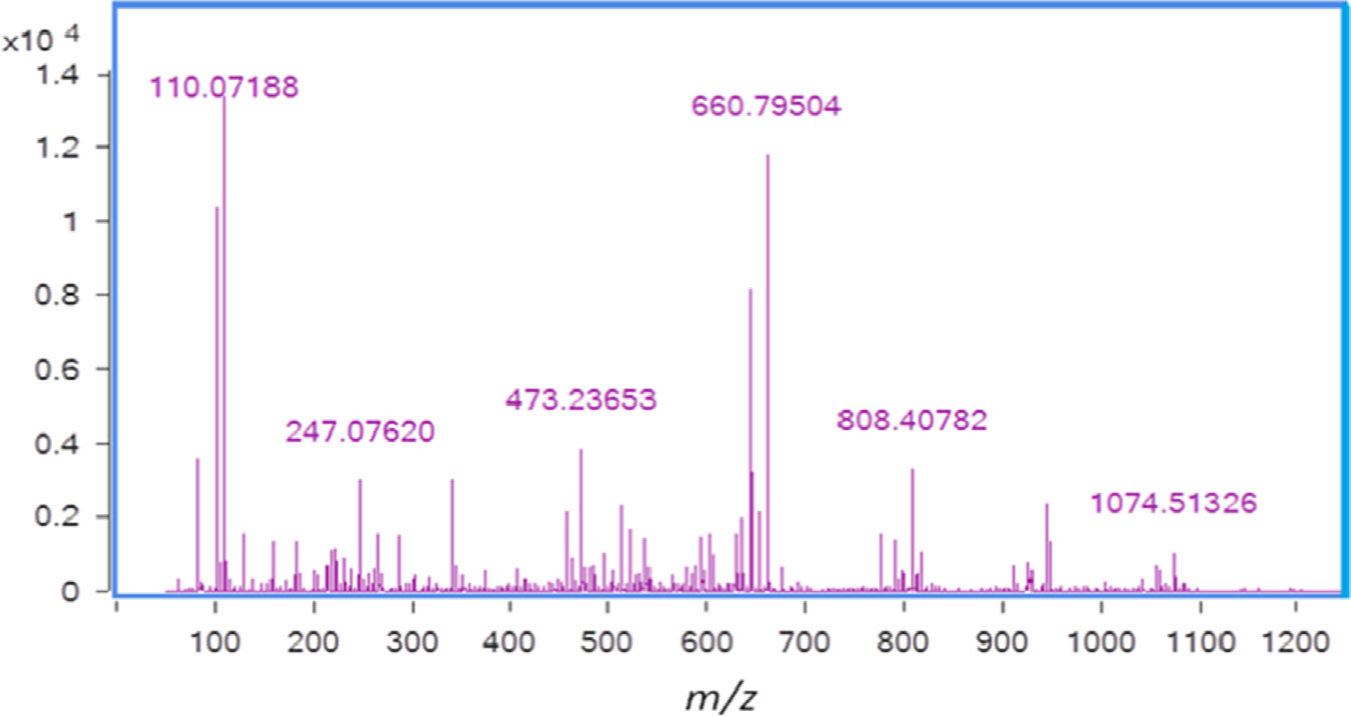
MS/MS spectra of doubly charged ACTH (4–11) with both amine labeled (*m*/*z*=660.7) by cleavable ester linked, NHS ester modified SiO_2_@Fe_3_O_4_ MNPs.

The mass spectra of a doubly charged, both amine modified ACTH (4–11) is shown in Fig. 3. The b and y ion were manually calculated and matched with major peaks in the spectra.

### 5.6 Mass spectrometric analysis of labeled ACTH (4–11), BSA and β-lactoglobulin

Chromatographic separation was performed by using a chip consisting of a 160 nL enrichment column and a 150 mm analytical column packed with C18, 5 m beads with 300 Å pores. The sample (2 μL) was transferred to the enrichment column via the capillary pump. Capillary pump was operated at a flow rate of 4 μL/min. The flow rate of nano pump was set to 600 nL/min. The MS source was operated at 300 °C with 5 L/min N_2_ flow and a fragmentor voltage of 175 V. Quad and TOF were operated in the positive ion mode. The calibration standards contained reference compounds of 322.048121 and 1221.990637 Da, which were continually released into the source for mass calibration. LC chromatograms and mass spectra were analyzed using Mass-Hunter software (Version B.0301; Agilent Technologies).

### 5.7 Quantification of active NHS ester groups on the surface of SiO_2_@Fe_3_O_4_ MNPs using ‘depletion’ approach

**Fig. 4:**
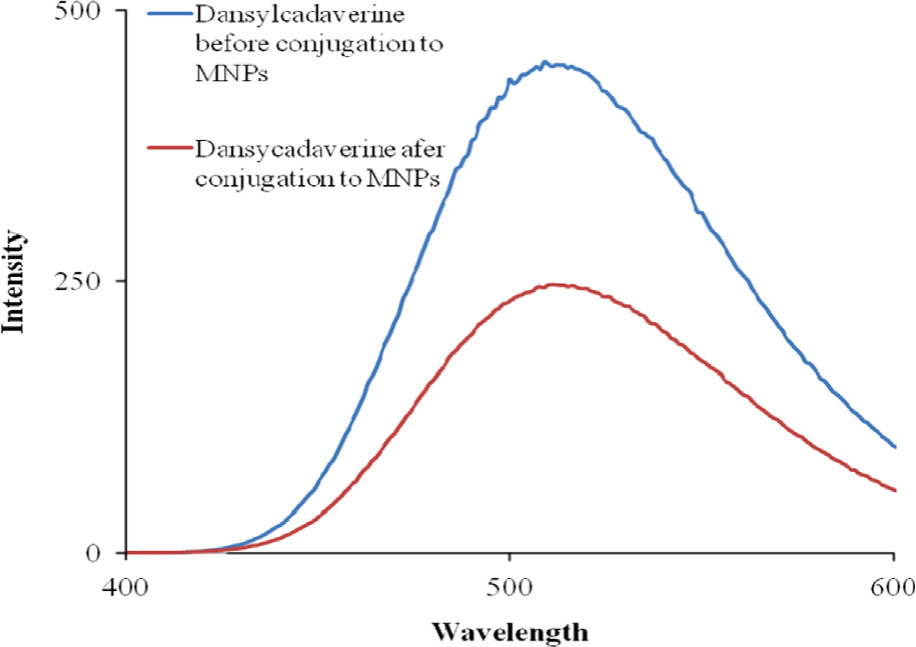
Fluorescence spectra of danylcadaverine before conjugation to cleavable ester linked NHS ester modified SiO_2_@Fe_3_O_4_ MNPs (blue) and danylcadaverine after conjugation to cleavable ester linked NHS ester modified SiO_2_@Fe_3_O_4_ MNPs (red).

Cleavable ester linked, NHS ester modified SiO_2_@Fe_3_O_4_ MNPs were prepared as shown in Scheme 1. Dansylcadaverine (700 μL of 20 μM) was mixed with cleavable ester linked, NHS ester modified SiO_2_@Fe_3_O_4_ MNPs (1 mg) for 40 min. The dansylcadaverine conjugated SiO_2_@Fe_3_O_4_ MNPs were magnetically separated, and the supernatants were collected for quantitative fluorescence measurements (Fig. 4). The quantity of conjugated dansylcadaverine was determined by subtracting the quantity of remaining dansylcadaverine after conjugation to NHS ester modified SiO_2_@Fe_3_O_4_ MNPs from the initial quantity of dansylcadaverine.
